# The Effect of a Multidisciplinary Trauma Team Leader Paradigm at a Tertiary Trauma Center: 10-Year Experience

**DOI:** 10.1155/2020/8412179

**Published:** 2020-08-13

**Authors:** Olivier Lavigueur, Joe Nemeth, Tarek Razek, Nisreen Maghraby

**Affiliations:** ^1^Université de Montréal, Montréal, Canada; ^2^Department of Emergency Medicine, McGill University, Montréal, Canada; ^3^Department of Trauma, McGill University, Montréal, Canada; ^4^Trauma and Disaster Medicine, Immam Abdulrhman Bin Faisal University, Dammam, Saudi Arabia

## Abstract

**Background:**

To illustrate the impact of the implementation of a multidisciplinary TTL program in 2005 on the mortality of trauma patients in a level 1 trauma center as well as admission rates and length of stay.

**Methods:**

Retrospective observational study of all trauma patients included in the provincial trauma database at the Montreal General Hospital between 1998 and 2015. The primary outcome studied was in-hospital mortality. The secondary outcomes studied were hospital and intensive care unit (ICU) rates of admission and hospital and ICU length of stay.

**Results:**

24,107 patients were included. We observed a statistically significant reduction in mortality of 1.25% or a relative reduction of 16% (*p* value = 0.0058; rate ratio 0.844 (95% CI 0.747–0.952)). ICU admissions were also significantly reduced where we observed a statistically significant absolute reduction of 4.46% or a relative reduction of 14% (*p* value = 8.38 × 10^−7^; rate ratio 0.859 (95% CI 0.808–0.912)). The ICU length of stay was increased by 0.91 days or 19.03% (*p* value = 0.016 (95% CI 0.167–1.655)). There was no observed change in overall length of stay (13.97 days pre-TTL and 12.91 post-TTL (*p* value = 0.13; estimate −1.053 (95% CI −2.424–0.318))).

**Conclusions:**

This article suggests that multidisciplinary TTL model may be beneficial in the care of trauma patients. Further subgroup analysis may help determine which patients could benefit more.

## 1. Background

### 1.1. Trauma, Trauma Centers, and the Early Years of the Montreal General Hospital Trauma Program

Trauma is the leading cause of death between the ages of 1 and 46 in the USA as well as the leading cause of years of life lost [1]—a trend that has not changed in over 20 years. In Canada, preventable injuries reflect the same reality. Every day, it is estimated that roughly 10,000 Canadians are injured and require medical attention. After assessment in the emergency department, 6% of those daily injured patients will be admitted to the hospital, 1.6% are left with disabling morbidity, and 0.4% will die [2]. Faced with the important mortality and morbidity associated with trauma in Canada, it is understandably crucial to continue improving the care of trauma patients. The aim of this article is therefore to assess the impact of the Trauma Team Leader (TTL) program by comparing the time period before and after its implementation at the Montreal General Hospital (MGH) as well as providing a possible explanation to the success of this paradigm in the hopes that other institutions may benefit from our experience over the years.

The implementation of a regionalized trauma system in the province of Quebec started in the early 1990s. At that time, the need to improve trauma care had become well recognized at the governmental level. A study performed before the implementation of trauma centers showed that trauma patients had an increased mortality compared to the results of the Major Trauma Outcome Study [3]. Another study looking at the Quebec trauma system at the time also found that physician on-scene had no significant impact on mortality but that mortality was lower if the patient had been brought to a hospital with specialized trauma services, especially if this was done under 60 minutes [4]. With supporting data coming from academic centers and from the Régie de l'assurance automobile du Quebec (RAAQ, now Société de l'Assurance Santé du Quebec—SAAQ) as well, the need to develop a regionalized trauma system became apparent and was undertaken. By 1993, four Quebec hospitals (Charles-Lemoyne (Charles-Lemoyne has since then changed its trauma center certification to level 2 with further centralization at the MGH), Quebec city's Enfant-Jesus, Sacré Coeur, and the Montreal General) were designated as the main trauma centers—“level 1” following the original concepts of the American College of Surgeons-Committee on Trauma (ACS-COT) guidelines [3, 5].

Simultaneously, emergency pre-hospital services were being formally organized in the province with the creation of the Corporation d'Urgence Santé in 1988 (the EMS service covering the territory of the cities of Montreal and Laval) [5]. Recognizing the importance of efficient prehospital care and transport in trauma [6], this gave an opportunity to more readily integrate EMS services within the trauma system being created. Indeed, before the creation of the trauma services in Quebec, EMS care of trauma victims involved transporting the patient to the nearest emergency department regardless of severity of injury or the hospital's ability to care for such patients [3]. In addition, for severe trauma, a doctor when available would be brought to the scene to procure Advance Life Support (ALS) treatments on-site which were out of the scope of practice of the EMS providers. A study by Sampalis et al. showed that trauma victims with delayed access to trauma centers in Quebec had greater mortality rates [7]. This deleterious practice in the care of trauma patients was eventually modified in 1995 in order to prioritize direct transfer to level 1 trauma centers where specialized care could be provided in a timely fashion [3, 8].

The results of the implementation of the Quebec trauma center system showed important improvement in mortality outcomes. Indeed, an initial study looked at the mortality of trauma patients in the Montreal region before (1987) and after (1993) the implementation of regionalized trauma care. Despite having similar injury severity scores (ISS) and after adjusting for variables such as age, gender, and mechanism of injury, the 1987 cohort was more likely to die than the 1993 cohort with a 3.25 relative risk ratio. This benefit increased proportionally to the ISS [9]. As level 2 and level 3 hospital designations were implemented in 1995-1996, newer prehospital triage and transfer protocols were created in order to favor adequate patient allocation. With these new measures in place, comparative analysis showed a decrease in mortality from 52 to 18% [10]. In fact, ever since the implementation of the trauma center designations and regionalization of trauma care in Quebec, data has shown an important decrease in mortality between 1991-1992 and 2001-2002 from 51.8% to 8.6% [3].

### 1.2. Trauma Team Paradigm

Several studies have shown the utility of a TTL program. Using a TRISS analysis (a tool trying to determine the probability of a survival based on ISS and the Revised Trauma Score—RTS), a study investigating an early trauma team implementation demonstrated that patients with higher TRISS scores had better outcomes if the trauma team had been activated [11]. Implementation of a TTL program has resulted in an absolute mortality reduction of 1.9% in all patients and 8.3% in patients with an ISS greater than 25 [12]. This is also observed in hospitals not recognized as trauma centers that have nevertheless developed a TTL program [12]. Other studies have shown that the implementation of trauma teams has halved the resuscitation time [13] and has shown, in pediatric settings, a tenfold decreased mortality [14] and decreased delayed injury diagnosis [15]. The odds ratio of mortality in a subgroup of trauma patients who were incorrectly triaged not to be treated by a trauma team was 7.6 compared to a similar group which benefitted from trauma team care [16].

The makeup of a trauma team is different from one institution to the next; notwithstanding its composition, its members should be skilled and knowledgeable enough to adequately manage the initial resuscitation of a severely injured patient in a horizontal approach. Considering the broad spectrum of injuries a severe trauma patient can suffer, the expertise of the trauma team members must also be broad which requires a multidisciplinary composition of the group. All life-saving interventions should therefore be in the skill set of the trauma team and its members should thus be found either in-house or within 15 minutes from the hospital [17]. The ACS-COT states that a high-level trauma team usually includes the following [17].

The trauma team leader plays the role of a guide and facilitator for all the other members of the team. The leader ensures that each phase of care flows in continuity to enhance the functioning of the trauma team [17]. Although the American College of Surgeons argue that the team should be led by a surgeon [17], this view is not substantiated with evidence and others believe anyone trained in trauma management can provide adequate care and the role of TTL can safely be rotated between various specialists [18–21]. A Canadian study designed specifically to find out if there was a difference depending on the background specialty concluded that surgeons, on call emergency physicians (EPs), or on-shift EPs can act as the TTL without a negative impact on patient survival or emergency department (ED) length of stay [22]. Another study also had similar conclusions about the impact of nonsurgical TTLs [18].

In Canada, the Trauma Association of Canada was established in 1983 [23] and released, in 1993, guidelines for trauma care based on the ACS position statement of 1981. These guidelines focused on the inclusiveness of trauma care rather than specifying trauma team composition (see Tables 1–3).

### 1.3. TTL Implementation at the MGH

Prior to 2005, the MGH trauma team did not function with a dedicated TTL. Initial management and resuscitation would be led by the emergency physician which would then consult and transfer to the appropriate services. The type of injuries and their severity would determine the admission service. In order to facilitate and centralize continuity of care, the TTL program was instituted in 2005. It began with six physicians from emergency medicine, two from trauma surgery, and one from anesthesiology for a total of nine. Two physicians had completed dedicated trauma fellowships beforehand while the others had not. There was no additional trauma-specific training completed by the TTL group afterwards. Establishing a dedicated group of physicians as TTL enabled them to be more exposed to the leadership role in trauma and accumulate and share their expertise with each other.

## 2. Methods

The MGH is one of two level 1 trauma centers in the Montreal area which sees approximately 10,000 ED visits for injury and approximately 1,500 admitted trauma patients annually over the last 10 years. Patients within the MGH catchment area who have suffered an injury are brought to the emergency department and the trauma team, led by a trauma team leader, becomes activated if specific criteria are fulfilled.

Trauma data in Quebec is prospectively collected into a province-wide registry. Inclusion criteria into the registry require one or more of the following: death resulting from injury, admission to non-intensive care unit (ICU) ward for 3 days or more, admission to an intensive care unit, or interhospital transfer. Inclusion into the MGH trauma database therefore follows this format. This study analyzed retrospective data from the MGH trauma database collected before (April 1998 to March 2005) and after (April 2005 to March 2016) the implementation of the TTL program. Ethical approval for this retrospective before and after study was obtained through the Research Institute of the McGill University Health Center.

Adult trauma patients who died from their injuries at any time, were admitted to an ICU at any time, were admitted 3 days or more under the MGH trauma service in a non-ICU ward, or were transferred from another institution were included in the trauma database. Metrics such as mortality, ISS, length of stay, age, sex, type of trauma, and type of care required were collected.

## 3. Results

**Figure 1:**
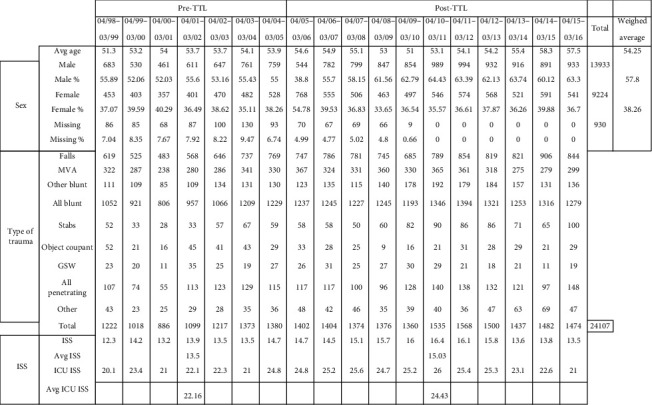
Patient demographics.

Patient data was collected from April 1998 to March 2016. A total of 24,107 patients fulfilled the inclusion criteria and were included in the study. There was a slight majority of males (57.8%) in our population with an average age of 54. The type of trauma requiring admission seen at our center is mostly blunt injuries (falls, followed by MVCs, and other blunt injuries). On the other hand, penetrating trauma represents a small portion of our patient population. The average injury severity score (ISS) increased from a 13.5 average before the implementation of the TTL program to 15.03 afterwards. Likewise, the average ICU ISS increased from 22.16 before to 24.43 after the TTL program implementation (Figure 1).

**Figure 2:**
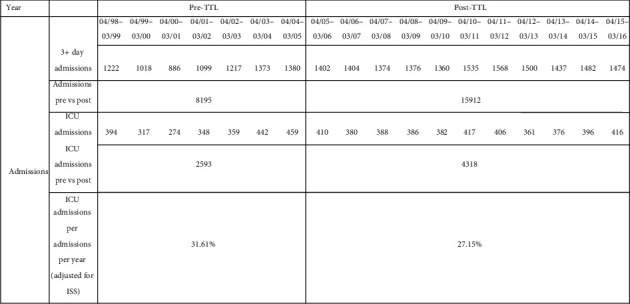
Admissions (ICU, more than 3 days in a non-ICU ward under the trauma service, transferred from another institution to the trauma service).

With regard to admissions during the period of the study, 8,195 patients were admitted (ICU, more than 3 days in non-ICU ward, or transferred from another institution to the trauma service) before the implementation of the TTL program, of which 2,593 were admitted to the ICU. 15,912 patients were admitted after the implementation of the TTL program and 4,318 of them were admitted to the ICU (Figure 2). When adjusting for the ISS using a Poisson statistical model, there was a 31.61% admission rate to ICU before the TTL program and a 27.15% rate after. This corresponds to a statistically significant absolute reduction of 4.46% or a relative reduction of 14% (*p* value = 8.38 × 10^−7^; rate ratio 0.859 (95% CI 0.808–0.912)) (Figure 2).

**Figure 3:**
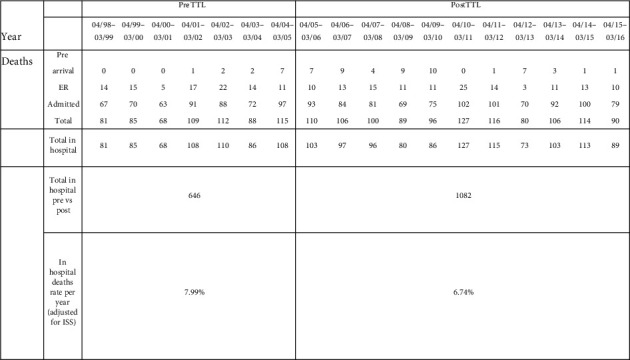
Mortality.

With regard to mortality, we accounted for deaths that only occurred within the hospital as the implementation of a TTL would have logically no impact on prehospital mortality. Thus, 646 patients died in the pre-TTL period compared to 1,082 in the post-TTL period. Again, using a Poisson statistical model, the ISS-adjusted in-hospital mortality rate was 7.99% pre-TTL and 6.74% post-TTL. This corresponds to a statistically significant absolute reduction of 1.25% or a relative reduction of 16% (*p* value = 0.0058; rate ratio 0.844 (95% CI 0.747–0.952)) (Figure 3).

**Figure 4:**
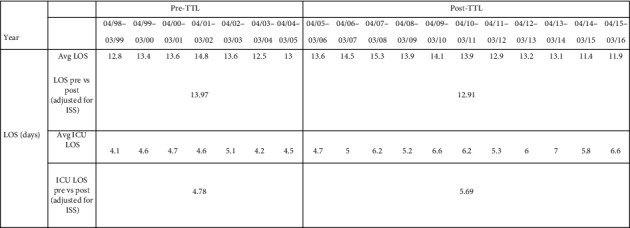
Length of stay.

A normal statistical model was used to investigate length of stay (LOS). The ISS-adjusted average LOS was 13.97 days pre-TTL and 12.91 post-TTL. There was no statistically significant difference between the two (*p* value = 0.13; estimate −1.053 (95% CI −2.424–0.318)). On the other hand, there was a statistically significant increase in ISS-adjusted ICU LOS from 4.78 days pre-TTL to 5.69 days post-TTL: an increase in 0.91 days or 19.03% (*p* value = 0.016; estimate 0.911 (95% CI 0.167–1.655)) (Figure 4).

### 3.1. Limitations

There were several limitations to our study. Firstly, the retrospective nature of the study does not allow us to establish direct correlation between the implementation of the TTL program and our outcomes. It is therefore difficult to determine if the results reflect the implementation of the TTL program or rather an improvement in overall trauma care. This is a single-center study with a particular population (predominance of blunt trauma) that may not reflect the population at other centers.

Lastly, the retrospective nature of the study also makes part of our results vulnerable to selection bias for not having included patients admitted to non-ICU wards for less than 3 days (although trauma patients transferred from another institution were included regardless of timing). The outcomes affected by this specific bias would be the secondary outcomes of overall admission rates and overall LOS. On the other hand, mortality and ICU specific outcomes would not have been affected.

## 4. Discussion

The MGH trauma team is always led by staff physicians. Additionally, the TTL coordinates the care of trauma patients with all medical specialties in the hospital as well as the paramedical specialties such as social workers, speech therapists, physiotherapists, or nutritionists to name a few.

The impact of the trauma program also extends beyond the peri-injury time. A lot of effort is dedicated to trauma prevention via the different community partners of our catchment area. Once a patient is discharged from our service, long-term rehabilitation care including a dedicated traumatic brain injury clinic helps our patient reintegrate society and recover as much of their pre-injury functionality as possible.

We have shown that the implementation of the TTL program at the MGH in 2005 was associated with a statistically significant decrease in mortality (1.25% absolute reduction, 16% relative reduction) and ICU admissions (4.46% absolute reduction, 14% relative reduction) despite no change in the overall length of stay. The ICU length of stay however was increased by 0.91 days.

We did not perform a subgroup analysis to determine whether the aforementioned outcomes were observed in a specific group (higher ISS, type of trauma) or in the entire population.

Due to the retrospective nature of the study, it was not possible to establish correlation between the exposure (TTL program implementation) and our measured outcomes. However, it would be very difficult to perform a randomized controlled trial (TTL vs no-TTL) for both logistical and ethical reasons.

To our knowledge, this article is the first to provide evidence suggesting mortality benefit in a mixed-specialists TTL program. The horizontal approach to trauma care in our team led by complementing specialists contributing their specific knowledge and know-how may help in the proper identification of injury severity, complexity, and priority, thus allowing the adequate allocation of patients to the appropriate resource in a timely fashion. Centralizing the TTL role to a smaller group also allows increased exposure for these individuals and improves their experience as leaders.

The pre- and post-TTL implementation periods in this study have different lengths—seven years and eleven years, respectively. One would expect leaders to perform better with time as they accumulate experience. Perhaps the better outcomes from the post-TTL period may be confounded by improved experience of the leaders in the latter years of a longer observation period. However, three new physicians joined the TTL group after 2005, lowering the average experience of the group. Even if experience had a major role in our findings, centralizing trauma leadership through TTL allows the group to accumulate and benefit from each other's experience more efficiently compared to a non-TTL group. The better outcomes observed in the post-TTL implementation group are then more likely to be due to the re-organization of the system with TTL rather than accumulated experience of individual members.

We submit that the MGH TTL program may be responsible for the positive outcomes observed since its implementation. On the other hand, perhaps continuing improvements in overall trauma care globally could have explained the improved outcomes over the years. Yet, possible evidence of the success of our model has been demonstrated in a recent article by Moore et al. which documented the mortality rates in Canada in trauma centers between 2006 and 2012 [24]. During this study period which coincides with the first seven years of our TTL program, the MGH had the lowest trauma mortality rate in Canada. Of note, this includes the other two trauma centers in the province of Quebec which operate within the same regionalized trauma system. One of these centers is also located in Montreal and treats a very similar trauma population. Considering we observed a possible mortality benefit after the TTL program implementation in 2005 and that our center performed the best in Canada from 2006 to 2012, this therefore suggests that our multidisciplinary TTL program model—unique in Canada—may in fact be the explanation behind our positive outcomes and not just due to overall improvement in trauma care. We did not compare our results to US trauma centers because of the difference in our populations with a significant predominance of blunt injury in our center.

## 5. Conclusion

The period of this particular trauma team leader paradigm was associated with decreased mortality and decreased ICU admission rate but increased ICU length of stay compared with the previous one. Perhaps the TTL model allows for the proper identification of traumatic severity and encourages the patients to be allocated to the correct resources more adequately.

A future direction to take following this article would be to determine if there are specific groups that benefit more from the TTL program approach by performing a subgroup analysis. The results of such a study would perhaps allow for increased precision of trauma team activation protocols.

While the potential impacts of this paper are encouraging, it is crucial to highlight that trauma care works best in the framework of an established and efficient trauma center system which involves prevention, prehospital emergency services, and adequate postdischarge convalescence/rehabilitation care.

## Figures and Tables

**Table 1:** ACS-COT composition of a trauma team.

Medical personnel	Nonmedical personnel
General surgeon	Laboratory technician
Emergency physician	Radiology technologist
Surgical and emergency residents	Security officers
Emergency department nurses	Chaplain or social worker
Critical care nurse	Scribe
Anesthesiologist or certified registered nurse anesthetist	
Operating room nurse	

**Table 2:** MGH trauma activation criteria.

Urgent trauma team activation	Nonurgent trauma team activation
Need for airway management (with significant mechanism or difficult airway)	Traumatic intracranial bleed or basilar skull fracture

Systolic BP < 90 in the ED	GCS < 10 in the ED (excluding MVC mechanism)

Penetrating injury to the head, neck, or trunk	Evidence of spinal cord injury

Mangled extremity or amputation above wrist or ankle	Unstable spinal cord injury

Need for blood transfusion in the resuscitation bay	Wide mediastinum with a significant mechanism of injury

Paralysis	Blunt abdominal trauma with tenderness

Burn >20% body surface area	Significant injury to a single system:
	(i) Solid organ injury on CT scan
	(ii) Flail chest or multiple rib fractures

Trauma transfer accepted by TTL (at their discretion)	Injuries to two or more body regions

ED physician may activate the trauma team at their discretion	Pelvic fractures
(i) Based on their initial assessment	
(ii) If they are unable to attend to the trauma patient due to increased workload in the resuscitation bay	

	Femoral fractures (except isolated hip fractures)
	Proximal extremity gunshot wounds
	Pregnant trauma patient at >20 weeks' gestational age
	Thoracoabdominal injury with an expected need for admission
	ED physician may also consult the trauma team at their discretion

**Table 3:** Physician composition of the present MGH TTL team.

Physicians	Residents/trainees
6 trauma surgeons	1–3 trauma surgery fellows
5 ER physicians	1–3 trauma/emergency medicine fellows
1 anesthesiologist	Rotating senior and junior residents from various specialties (usually surgical)
	Elective medical students

## Data Availability

The MGH trauma database from 1998 to 2016 used to support the findings of this study is available from the corresponding author upon request.
